# Zinc Concentration Dynamics Indicate Neurological Impairment Odds after Traumatic Spinal Cord Injury

**DOI:** 10.3390/antiox9050421

**Published:** 2020-05-13

**Authors:** Raban Arved Heller, André Sperl, Julian Seelig, Patrick Haubruck, Tobias Bock, Theresa Werner, Albert Besseling, Qian Sun, Lutz Schomburg, Arash Moghaddam, Bahram Biglari

**Affiliations:** 1 Institute for Experimental Endocrinology, Charité—Universitätsmedizin Berlin, Corporate Member of Freie Universität Berlin, Humboldt-Universität zu Berlin, Berlin Institute of Health, 13353 Berlin, Germany; julian.seelig@charite.de (J.S.); qian.sun@charite.de (Q.S.); lutz.schomburg@charite.de (L.S.); 2 Heidelberg Trauma Research Group, Department of Trauma and Reconstructive Surgery, Center for Orthopedics, Trauma Surgery and Spinal Cord Injury, Heidelberg University Hospital, 68118 Heidelberg, Germany; andre.sperl@gmx.de (A.S.); patrick.haubruck@sydney.edu.au (P.H.); tobias.bock@outlook.com (T.B.); 3 Raymond Purves Bone and Joint Research Laboratories, Kolling Institute of Medical Research, University of Sydney, Sydney 2065, NSW, Australia; 4 BG Trauma Centre Ludwigshafen, Department of Physiotherapy, 67071 Ludwigshafen, Germany; theresa.werner@bgu-ludwigshafen.de (T.W.); albertus.besseling@bgu-ludwigshafen.de (A.B.); 5 Aschaffenburg Trauma and Orthopedic Research Group, Center for Orthopedics, Trauma Surgery and Sports Medicine, Hospital Aschaffenburg-Alzenau, 63739 Aschaffenburg, Germany; arash.moghaddam-alvandi@klinikum-ab-alz.de; 6 BG Trauma Centre Ludwigshafen, Department of Paraplegiology, 67071 Ludwigshafen, Germany; bahram.biglari@bgu-ludwigshafen.de

**Keywords:** traumatic spinal cord injury, zinc, neuroprotection, neurotrauma, regeneration

## Abstract

Traumatic Spinal Cord Injury (TSCI) is debilitating and often results in a loss of motor and sensory function caused by an interwoven set of pathological processes. Oxidative stress and inflammatory processes are amongst the critical factors in the secondary injury phase after TSCI. The essential trace element Zinc (Zn) plays a crucial role during this phase as part of the antioxidant defense system. The study aims to determine dynamic patterns in serum Zn concentration in patients with TSCI and test for a correlation with neurological impairment. A total of 42 patients with TSCI were enrolled in this clinical observational study. Serum samples were collected at five different points in time after injury (at admission, and after 4 h, 9 h, 12 h, 24 h, and 3 days). The analysis of the serum Zn concentrations was conducted by total reflection X-ray fluorescence (TXRF). The patients were divided into two groups—a study group S (*n* = 33) with neurological impairment, including patients with remission (G1, *n* = 18) and no remission (G0, *n* = 15) according to a positive AIS (American Spinal Injury Association (ASIA) Impairment Scale) conversion within 3 months after the trauma; and a control group C (*n* = 9), consisting of subjects with vertebral fractures without neurological impairment. The patient data and serum concentrations were examined and compared by non-parametric test methods to the neurological outcome. The median Zn concentrations in group S dropped within the first 9 h after injury (964 µg/L at admission versus 570 µg/L at 9 h, *p* < 0.001). This decline was stronger than in control subjects (median of 751 µg/L versus 729 µg/L, *p* = 0.023). A binary logistic regression analysis including the difference in serum Zn concentration from admission to 9 h after injury yielded an area under the curve (AUC) of 82.2% (CI: 64.0–100.0%) with respect to persistent neurological impairment. Early Zn concentration dynamics differed in relation to the outcome and may constitute a helpful diagnostic indicator for patients with spinal cord trauma. The fast changes in serum Zn concentrations allow an assessment of neurological impairment risk on the first day after trauma. This finding supports strategies for improving patient care by avoiding strong deficits via adjuvant nutritive measures, e.g., in unresponsive patients after trauma.

## 1. Introduction

Traumatic spinal cord injury (TSCI) is considered as one of the most severe injuries in traumatology. As it predominantly affects young patients [1,2], it requires a lot of experience, practice, and knowledge to assure the best possible care and increase the chances of a fortunate outcome for the patient. The physical and psychosocial [3] consequences for patients are debilitating as well as the economic consequences, which are a concern for the patient, his or her environment, society, and health care institutions [4]. Traumatic damage to the spinal cord proceeds in several sequential phases. The primary injury phase is defined by the immediate mechanical damage to the tissue mostly due to shearing, laceration, acute stretching, and sudden acceleration-deceleration events causing spinal shock [5,6]. Hereafter, a more prolonged secondary injury phase is set in motion that involves complex inflammatory responses. Finally, the chronic phase is characterized by autonomic dysregulations [6,7,8,9].

Despite the growing acceptance of early surgery post SCI [10], detailed anamnesis and thorough clinical examinations at the time of admission to the hospital can be difficult, as patients might be unresponsive due to coma or pre-clinical sedation. An objective diagnostic marker that is capable of indicating the risk for neurological impairment within the first 24 h would be of high importance for enabling the rapid, adequate, and most promising personalized therapeutic decisions.

Zinc (Zn) is one of the essential trace elements in the human body that plays a fundamental role in a variety of biochemical pathways, in proteins and enzymes, and as a structural element, as well as a local signaling molecule with particular relevance for the immune system [11]. Zn is particularly essential in the early stages of immune response, e.g., by supporting the activity and efficiency of natural killer cells [12]. The usual range of Zn in human blood lies between 650 and 1100 µg/L (i.e., 10.0–17.0 µmol/L) [13], and the leading cause of zinc deficiency is malnutrition. Many critical processes of the immune response depend on Zn availability, e.g., (i) the formation of extracellular traps by neutrophils, (ii) shifting the balance from humoral immune responses to cell-mediated immunity, and (iii) dynamically adapting the inflammatory response to avoid overshooting immune cell activities. The injury mechanism of TSCI is reported to strongly activate the TLR4/NF-κB signaling pathway in monocytes [14,15]. A sufficiently high Zn supply seems to contribute to an adequate activation of NF-κB [16] and downstream target genes, thereby finetuning the immune response dynamically. There are two main types of transport proteins which are responsible for Zn homeostasis. The Zn transporters ZnT are mainly responsible for Zn effluxes, and the Zip family of transporters are responsible for the increase in cytoplasmic Zn [17]. NF-κB has been reported to bind to the ZIP8 promoter and thus to be associated with the Zn uptake in monocytes [18].

Known as an acute-phase reactant because of its rapid redistribution from blood-serum into cellular compartments [19], Zn is supposed to be an ideal marker, especially for the early phase of (trauma-induced) inflammation. Furthermore, Zn has also been implicated in neuronal damage associated with Traumatic Brain Injury (TBI), stroke, and seizure [17,20]. A decline in serum zinc levels has recently been documented in brain-injured patients [17,21]. The dynamics are not characterized in much detail and there is controversy about a possible neurotoxic or neuroprotective role of Zn after TBI [17].

The database on Zn status and time-dependent changes after SCI is sparse [13,22,23]. Last year, in 2019, Kijima et al. reported that the acute-phase changes in serum zinc concentration were inversely correlated with the long-term functional outcome after experimental SCI in a model system. This finding was attributed to activated infiltrating monocytes that actively accumulate Zn from extracellular sources. The interesting results on Zn dynamics were confirmed in an analysis of 38 cervically injured SCI patients, yielding a predictive model for a long-term functional outcome, i.e., the ability to walk [23].

Given these promising results, we decided to evaluate the model for its diagnostic suitability in TSCI. To this end, we analyzed the dynamic alterations in serum Zn during the first 72 h after injury in short intervals to examine the possible relation between the early changes of total serum Zn concentration with the presence of neurological impairment and the patient’s outcome. We hypothesized that the Zn concentrations are dependent on additional parameters such as the exact time after TSCI and the patients’ neurological levels of injury (NLI). Our results verify the notion of serum Zn as a promising diagnostic biomarker of TSCI and pinpoint the most informative time points for analysis and prediction.

## 2. Materials and Methods

The study had been approved by the local Ethics Committee of the University of Heidelberg (S514/2011) and was registered (Study-ID: DRKS00009917/Date of Registration: 23.03.2016/Universal Trial Number (UTN): U1111-1179-1620) at the German Clinical Trial Register (Deutsches Register Klinischer Studien—DRKS). Data collection and processing were performed according to good scientific practice. All the study participants signed an informed consent form and agreement to participate willingly and were informed that they could voluntarily choose to leave the study without any negative consequences. The manuscript was composed according to the TRIPOD statement (Transparent Reporting of a multivariable prediction model for Individual Prognosis or Diagnosis) [24].

### 2.1. Source of Data

**Figure 1:**
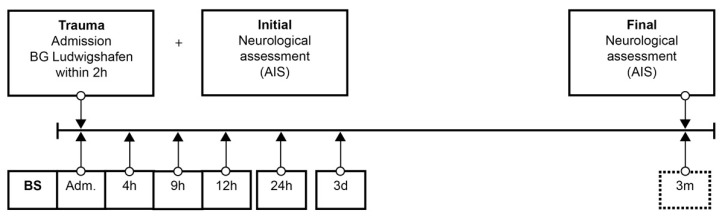
Standardized blood sample collection protocol. Abbreviations: BS = blood sample; Adm. = admission; AIS = American Spinal Injury Association (ASIA) Impairment Scale; h = hours; d = days; m = months.

Patient data were collected from the hospital database. The neurological level of injury (NLI) was defined as the lowest neurological level where both motor and sensory functions are intact. Analog to the duration of the study (72 h) in the protocol of Kijima et al. [23], from admission to the end of follow up, 6 blood samples were taken at specific points in time over 72 h according to our protocol (Figure 1). The protocol was adapted from our previous studies [25,26,27,28,29,30,31,32,33]. After blood sampling and 20 min of coagulation, the blood was centrifuged at 3000 rpm with an RCF (Relative Centrifugal Force) of 1008 m/s^2^, aliquoted, and stored at −80 °C until analysis. Missing samples in the protocol were mostly due to early and urgent interventions. Analyses were conducted in an S1 laboratory of the Charité Universitätsmedizin zu Berlin in accordance with §3 Biostoffverordnung (BioStoffV). Trace element concentrations were determined by a total reflection X-ray fluorescence (TXRF) analysis, essentially as described in [25,34]. Briefly, the serum samples were diluted 1:2 (*v*/*v*) with a Gallium solution as an internal standard and applied to polished quartz glass discs. After drying, a benchtop TXRF device (PicoFox S2, Bruker Nano, Berlin, Germany) was used for recording the fluorescence spectra emitted from the elements upon X-ray excitation. An internal laboratory quality control was included in each measurement run, and all the samples were measured in duplicate. Inter-assay coefficients of variation (CV) were typically below 10%, as determined with a commercial standard serum (Sero AS, Seronorm, Billingstad, Norway) [25,34].

### 2.2. Participants

**Figure 2:**
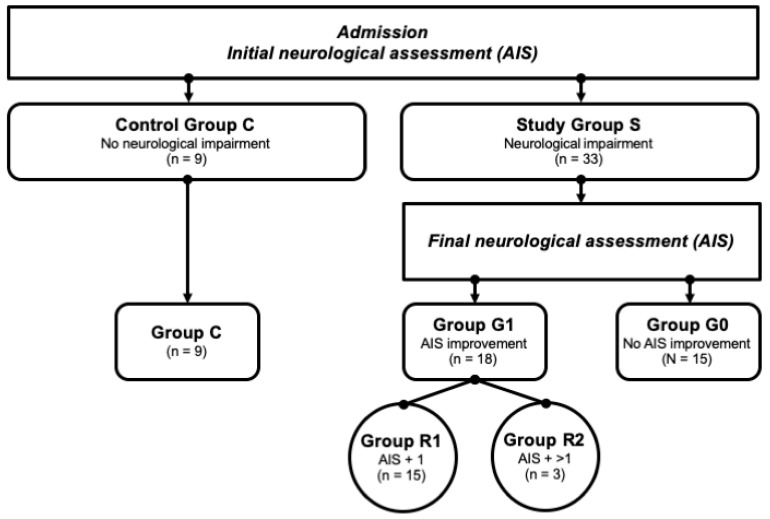
Patient collective identification scheme. Abb.: G0/G1 = patients with (G1) or without neurological remission (G0) after 3 months; AIS + 1/AIS + 2 = patients with a neurological improvement of 1 or 2 AIS levels within 3 months after injury.

In the Department of Paraplegiology at the BG Trauma Center Ludwigshafen, venous blood samples of traumatic spinal cord-injured patients were collected prospectively between 2011 and 2018. All the patients suffered from at least one fracture of the spine and were classified with respect to the AO (“Arbeitsgemeinschaft für Osteosynthesefragen”; German for “Association for the Study of Internal Fixation”) classification [35]. The exclusion criteria were non-traumatic spinal cord injury (SCI), traumatic brain injury, severe abdominal trauma, traumatic amputation of extremities, coma, or any additional life-threatening trauma apart from SCI. The participants were not given methylprednisolone sodium succinate during the study participation. The patients included in the current study were assigned to the study group S (*n* = 33) that consisted of two subgroups—G1 (*n* = 18) included patients with neurological remission, and G0 (*n* = 15) included patients without neurological remission. Nine subjects with vertebral fractures without neurological impairment were analyzed and served as the control group C (*n* = 9). All the patient characteristics are shown in Table 1. Additionally, patients in G1 were divided into 2 separate remission subgroups according to their AIS level increase: patients that presented an AIS conversion of 1 level formed the group R1 (AIS imp. = +1), and those with more than 1 level were allocated to group R2 (AIS imp. > +1). A flowchart detailing the patient allocation can be found in Figure 2.

### 2.3. Outcome

To classify the neurological impairment, AIS grades were determined by experienced examiners, according to the International Standards for Neurological Classification of SCI (ISNCSCI; see Table 2) [36]. Initial examinations (AIS initial) were performed within 72 h after admission in awake and responsive patients; final examinations (AIS final) took place 3 months after the trauma. Neurological remission was defined as an improvement in AIS grade within the 3 months after the trauma.

### 2.4. Predictors

The individual Zn concentration patterns were analyzed concerning the presence or absence of (1) neurological impairment, and (2) neurological remission/functional recovery.

### 2.5. Sample Size

The study enrolled 42 patients (30 male, 12 female).

### 2.6. Missing Data

The mean follow-up within the first 72 h was higher than 75%; missing values were excluded from the analysis via pairwise deletion [37].

### 2.7. Related Work

The register has already been used to address related scientific issues before in the quest for suitable biomarkers for this devastating disease [8,25,26,27,28,29,30,31,32,33,38,39,40,41]. The analyses were performed retrospectively, whereas the samples were collected prospectively in the biobank. Therefore, the particular analyses include different numbers of patients according to availability and the specific scientific issue. Thus, no double reporting of patient collectives occurred, although some patients were enrolled in more than one study when they met the inclusion criteria and a suitable sample was available.

### 2.8. Statistical Analysis

The non-parametric test methods were assessed to investigate the location shifts between groups. Categorical variables were evaluated using Fisher’s exact test. As this is an exploratory post-hoc analysis, all p-values are to be interpreted descriptively. No prior power analysis was carried out, as they may be of little value in early exploratory studies where scarce data are available on which to base the calculations [42], and no adjustment for multiple testing was adopted. All statistical tests use an α-level of 0.05, and statistical significance was defined as *p* > 0.05 (n.s), *p* < 0.05 (*), *p* < 0.01 (**), or *p* < 0.001 (***). Univariate logistic regression was utilized to assess the predictive potential of the variables. The model comparison was based on the Akaike information criterion (AIC). The primary measure for the predictive performance of the logistic regression model was the area under the curve (AUC) of the Receiver Operator Characteristic (ROC) analysis. All the statistical calculations were performed with R version 3.6.0 [43]. The figures were created using the package ”ggplot2” [44].

## 3. Results

### 3.1. Demographics

**Figure 3:**
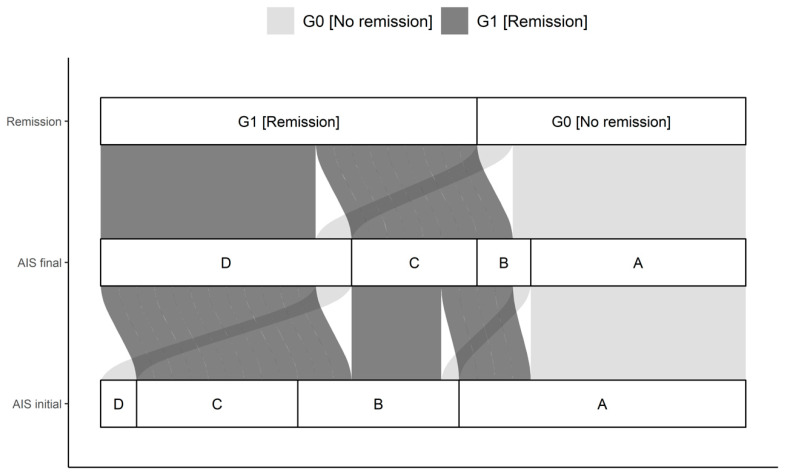
Distribution of initial AIS and final AIS in G0 and G1. The connections indicate the individual configuration of each patient with initial neurological impairment with respect to the initial and final AIS as well as the group assignment to either G0 or G1. Abb.: AIS initial = AIS level at admission; AIS final = AIS level 3 months after injury.

A total of 42 patients with a traumatic spinal cord injury were studied over 3 months. Thirty male and 12 female study participants were included. The median age of the patient cohort was 42.0 (IQR, 27.8-59.0) years, ranging from 15 to 78 years. The G0 and G1 differed significantly in their NLI (*p* = 0.035). Initial as well as final AIS grades differed significantly comparing G0 and G1 (*p* < 0.001), with more AIS patients classified as A in G0 than in G1. The complete overview is given in Table 1 and Figure 3.

### 3.2. Analysis of the Entire Patient Collective

**Figure 4:**
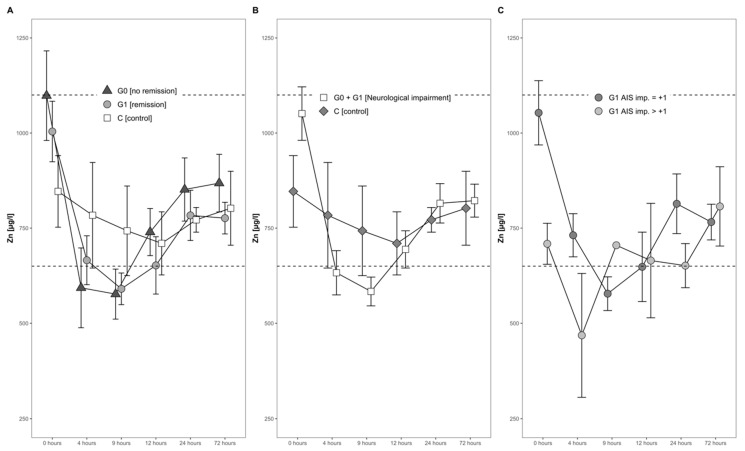
Mean Zn serum level comparison, presenting and comparing patients with and without neurological remission and controls (**A**) with neurological impairment, as well as controls (**B**) and patients with neurological remission that presented an AIS conversion of 1 level (G1 AIS imp. = +1) to those with an AIS level increase of more than 1 level (G1 AIS imp. > +1) (**C**). Expressed as mean values ± standard error of the mean. The Mann–Whitney U-Test assessed significant differences between both groups at each time point (*p* < 0.05).

The mean serum Zn levels dropped by almost 30% during the first 4 h after the injury, from an average of 952 µg/L to 676 µg/L. A minimum was reached 9 h after the trauma, when the average serum Zn concentration was slightly below the physiological range (650–1100 µg/L). Hereafter, a gradual and constant increase until 1 day after injury set in (Figure 4A,B).

### 3.3. Comparison of Serum Zn Concentrations in Relation to Neurological Remission

The concentrations of serum Zn in both the group of patients with remission (G1) and without remission (G0) dropped during the first 4 h, reaching the lowest concentration 9 h after admission. This decline was transient, and the concentrations in both groups increased gradually thereafter (Figure 4A). No significant difference was recorded throughout the time course.

### 3.4. Zn in Neurological Patients in Relation to Controls with Vertebral Fractures

The Zn dynamics in the combined group of neurological patients, i.e., S (G0 + G1), in comparison to the control group (C) differed in the first 24 h after injury. At admission, patients in S showed 28% higher concentrations of Zn, albeit to a non-significant extent (*p* = 0.150, S > C). Even though the Zn concentrations dropped in both groups during the first 4 h, the decline in group S was more distinct as compared to group C (decline of 35% versus 7%). Notably, while the levels in S fell on average under the reference range for serum Zn concentration, the control group C stayed within the reference range at all points in time. No significant difference was detected from admission to 24 h after injury (Figure 4B).

### 3.5. Comparison within the Group of Recovering Patients in Relation to Impairment

In patients with neurological remission, a distinct pattern of serum Zn concentrations was recorded, whereas in those with an AIS increase of more than 1 level, the concentrations of serum Zn were relatively low in the time frame of 4 h to 24 h after injury. In comparison to the initial Zn concentration, the AIS imp > + 1 patients reached their minimum of 58% at 4 h after trauma, whereas patients with an AIS imp = +1 reached their minimum of 56% at 9 h after the TSCI. The corresponding patterns are shown in Figure 4C.

### 3.6. Comparison of Zn Concentration Dynamics from Admission to 9 h after Injury

**Figure 5:**
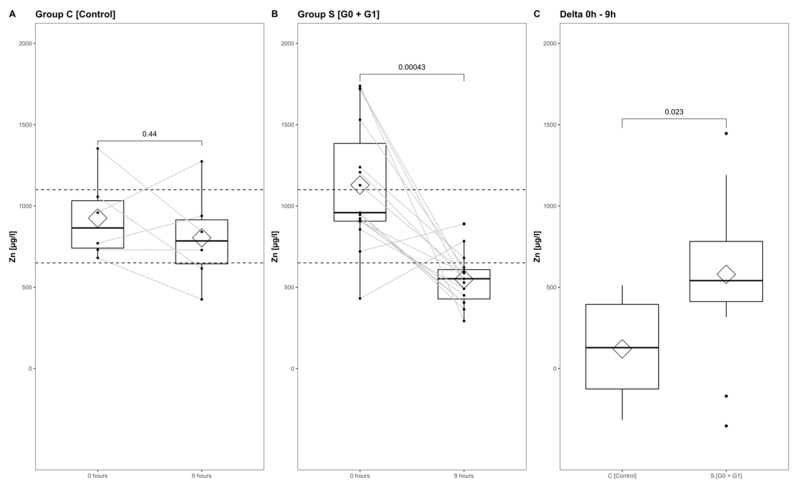
Zn serum level comparison of all patients at admission to 9 h after injury in group C (**A**) and group S (**B**), and from admission to 9 h after injury in group S and group C (**C**). The Wilcoxon signed-rank test assessed significant differences between groups, while the corresponding p-value is given above.

**Figure 6:**
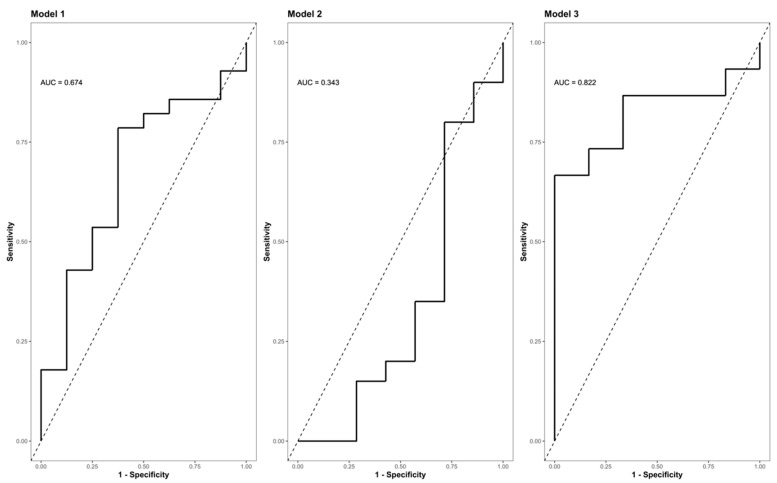
ROC (Receiver Operator Characteristic) curve analysis of the final model II indicating its AUC (area under the curve) and the corresponding confidence interval (CI) for predicting the presence or absence of neurological impairment as a function of the difference in the Zn concentration (0 h–9 h). Model I (S/C ~ Zn 0 h [µg/L]); model II (S/C ~ Zn 9 h [µg/L]); (S/C ~ Zn 0 h–9 h [µg/L]). Abb.: S/C = criterion presence or absence of a neurological impairment (study group S vs. control group C); Zn 0 h [µg/L] = Zn concentration at admission/0 h after injury; Zn 9 h [µg/L] = Zn concentration 9 h after injury; Zn 0 h–9 h [µg/L] = difference in the Zn concentration 0 h–9 h after injury; AUC = area under the curve.

The Zn concentrations in group S (consisting of G0 + G1) dropped significantly, reaching their minimum within the first 9 h after injury (*p* < 0.001) (Figure 5, part A indicates the development in group C; part B shows the corresponding dynamics in group S). Differences from admission to 9 h after injury were significantly more distinct in group S (G0 + G1) compared to group C (*p* = 0.023) (Figure 5C). A binary logistic regression analysis was performed to assess the predictive value of dynamic changes in the serum Zn concentration regarding its potential to identify patients with or without high risk for neurological impairment. The final model (Table 3, model 3) including the difference in the early alterations in Zn concentration (Zn 0 h–Zn 9 h) yielded an area under the curve (AUC) of 82.2% (CI: 64.0–100.0%) with regards to the occurrence of neurological impairment (Table 3, model 3; Figure 6, model 3). Compared to the univariate model 1 (S/C ~ Zn 0 h) and model 2 (S/C ~ Zn 9 h), we detected an improvement in AIC from 39.88 in model 1 to 32.16 in model 2, compared to 24.45 in the final model 3 (S/C ~ [Zn 0 h–Zn 9 h]). The Pseudo-R² (Cragg–Uhler) rose from 0.09 in model 1 to 0.14 in model 2 and finally to 0.29 in model 3, which also supports the eminently higher proportion of the total variability of the outcome that is accounted for by the model [45]. This assessment is also supported by the large effect size estimated via Cohen’s d [46] for the difference regarding the early alterations in Zn concentration.

## 4. Discussion

In the current study, we monitored the serum Zn concentration in short time intervals during the initial phase after TSCI in correlation to the outcome and neurological impairment. Our results support a possible association between the decreasing serum zinc concentrations and the presence of neuronal damage after the TSCI. Data from the current study indicate some considerable alterations in the circulating levels of the trace element Zn with potential relevance for a better initial diagnosis of neurological impairment, especially in unresponsive patients. Additionally, the prediction of the outcome might be improved for subgroups of patients with cervical TSCI. However, the transferability to patients with thoracic or lumbar TSCI seems to be limited. Furthermore, the underlying pathomechanisms contributing to the interaction need to be elucidated more specifically in further studies, as relevant covariates in the Zn concentration patterns of the acute phase after TSCI were identified.

Recently, Kijima et al. [23] described that the acute serum Zn concentrations decreased proportionally to the severity of the injury to the spinal cord in an SCI mouse model. Our study was intended to test this promising notion in TSCI. An important difference between the studies was the age of the patients analyzed, as they were significantly younger at admission (*p* = 0.004) in our study compared to the patients studied by Kijima et al. (Table 4) [23].

**Figure 7:**
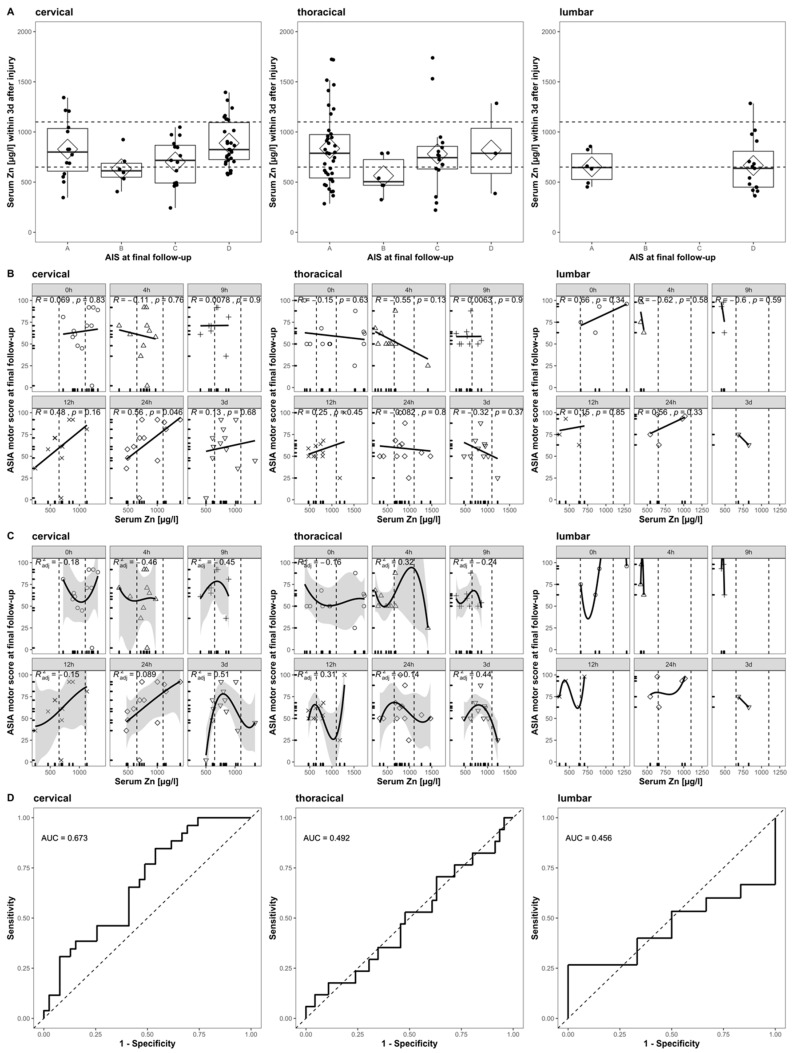
Comparison to Figure 4 in Kijima et al., 2019. The relation of acute serum zinc concentrations to the functional prognosis after SCI in humans. Each row (**A**–**D**) is divided into three columns according to the patient’s NLI (cervical, thoracic, and lumbar). (**A**) The admission serum zinc concentrations (pooled values from admission to 3 days after injury) of the patients with AIS at final follow-up. (**B**) Scatter plots illustrating the correlations between the ASIA motor score at final follow-up and the serum zinc concentration at admission (Pearson’s correlation coefficient) at admission, 4 h, 9 h, 12 h, 24 h, and 3 days after injury. (**C**) The results of a non-linear regression analysis of the functional outcomes using the serum zinc concentration at admission (formula *y* = a × 3 + b × 2 + c*x* + d), 4 h, 9 h, 12 h, 24 h, and 3 days after injury. (**D**) The receiver operating characteristics (ROC) curves of the prediction model based on the acute serum zinc concentrations (pooled values from admission to 3 days after injury) for discriminating between the ability to walk independently (with or without an assisting device) or not.

When applying the same inclusion and exclusion criteria to our cohort, we can form a subset (*n* = 14) for direct comparison. The reduced sample size is mainly due to the exclusion of patients with any other than cervical TSCI. With respect to the relationship between the initial Zn concentrations and the AIS at the final follow-up, the ASIA motor score at the final follow-up, and the patient’s ability to walk, a direct comparison indicates some congruent findings (Figure 7) and some differences. Notably, our extended time course and the analysis of patients suffering from thoracic and lumbar TSCI provides a more detailed picture (Figure 7, columns 2 and 3).

The correlation of poor outcome with low serum zinc concentrations at admission is observed in both studies and can be confirmed specifically for patients with a final AIS of category B, C, or D. Patients with a final AIS of category A presented a higher variance than others, while their median and mean Zn concentration was above those with AIS B or C (Figure 7A, cervical). The strong positive correlation between the serum zinc at admission and the ASIA motor score at the final follow-up is similarly observed in both studies. A time-dependent association between these two parameters is given, with the highest and most significant correlation at 24 h after injury (*R* = 0.56, *p* = 0.046) (Figure 7B, cervical). The overall correlation observed might be merely driven by and of particular relevance to a specific subgroup of patients with cervical TSCI and for the alterations in serum Zn that are detectable at the 24 h point in time after the injury. The non-linear regression failed to predict the functional outcome with sufficient significance. As the relation at any point in time is not logistic, we used a third-degree polynomial function with the best adj. The *R^2^* = 0.51 at 3 days after injury (Figure 7C, cervical). Evaluating the pooled serum Zn concentrations of the first 3 days was useful for distinguishing between those patients who were able to walk independently and those who were not (AUC = 67.3%, CI: 54.1–80.4%) (Figure 7D, cervical). While the relationship of the outcome and the initial serum Zn concentration presents a similar pattern in cervical and thoracically injured patients (Figure 7A, cervical and thoracic), the correlation and regression analysis reflects the highly time-dependent distribution of Zn concentrations (Figure 7B,C, cervical and thoracic). The final logistic regression for thoracically injured patients does not add any further predictive information (Figure 7D, thoracic). The sample size of lumbar injured patients is unfortunately minimal (*n* = 5), and the results presented are to be interpreted descriptively (Figure 7A–D, lumbar).

Regarding the molecular processes underlying the decrease in serum Zn concentrations after TSCI, we speculate that—according to the speed of changes—an active uptake into monocytes needs to be considered. In this respect, activated monocytes might contribute actively to the changes observed in peripheral serum [23], as a TSCI-induced activation of the TLR4/NF-κΒ/ZIP8 pathway is known to increase the Zn uptake in monocytes [14,15,18,23]. In parallel, the activation of the transcription factor NF-κΒ is associated with the polarization of monocytes into M1 and M2 [47], while the specific M1/M2 balance is orchestrated by the p50 subunit [48]. Meanwhile, the numerical presence of M1 or M2 macrophages is often thought to directly imply function (for example, kill or repair); such associations need not be true [47]. However, recent evidence suggests a significant beneficial effect from the M2 or “alternatively” activated macrophage phenotype [49,50,51,52,53]. Evidence regarding the influence of Zn on the specific polarization of monocytes in patients with TSCI remains scarce. Nevertheless, the overall activation of NF-κΒ is inhibited by ZIP8 activation, a transcriptional target of NF-κB, negatively regulating proinflammatory responses via the down-modulation of IkB kinase (IKK) activity [18]. An association of inhibited NF-κΒ activation via Zn administration with a favorable outcome in mice with TSCI has been confirmed by Li et al. in 2019 [54]. These findings open up the possibility of differential immune manipulation based on Zn metabolism as a therapeutic approach for patients with TSCI. Additionally, these processes might be supported by autophagy-enhancing drugs that positively affect multiple cell types, promoting neuron and oligodendrocyte survival, oligodendrocyte differentiation, and decreasing neuroinflammation through the modulation of the inflammatory polarization process via the inhibition of NF-κB [55].

The dynamical alterations in serum zinc observed in our study are supported by the literature and in line with several findings reported from animal models [56,57,58]. Besides the actual mechanic trauma, metabolic processes in the secondary injury phase after TSCI lead to astrocyte, oligodendrocyte, and neuronal cell death. Here, glutamate excitotoxicity is a frequently described mechanism for neuronal tissue death in this phase [59,60]. The available data indicate that reduced extracellular Zn levels following trauma contribute to the dysregulation of Nuclear Factor-κB (NF-κB), thereby enhancing the glutamate excitotoxicity of astrocytes and increasing the death of oligodendrocytes. The exposure of oligodendrocytes to glutamate causes toxicity under Zn-deficient conditions, but not in the presence of a sufficiently high Zn concentration, highlighting the importance of Zn for the prevention of glutamate excitotoxicity [61]. In 2002, Zhang et al. demonstrated that Zn inhibits charge currents through ionotropic glutamate receptors in a dose-dependent manner. They observed that at high extracellular glutamate concentrations (200 µM), low levels of Zn fail to inhibit Ca^2+^ currents through AMPA (α-amino-3-hydroxy-5-methyl-4-isoxazolepropionic acid) receptors.

Interestingly, supplemental extracellular Zn attenuated these currents in a dose-dependent manner [62]. To investigate the effects of free vesicular Zn, Doering et al. [63] conducted an experimental study in which damage and Zn movements were analyzed in an animal model of traumatic brain injury (TBI). ZnT3 KO mice were compared to wild-type (WT) mice after TBI and presented with elevated neuronal damage. These differences were nullified by the chemical inhibition of the vesicular Zn transport in WT mice, which resulted in similar cellular damage within the first 24 h.

Recent evidence, however, suggests that neurodegeneration, mediated via ER stress and the disruption of Ca homeostasis and following transient global ischemia such as TSCI, may underlie the mechanisms of Zn-induced neurotoxicity [64]. In ischemia conditions, a considerable amount of Zn (up to 300 μM) is co-related with glutamate into synaptic clefts by membrane depolarization. These conditions are suspected of harming neurons in the hypothalamus, as shown, e.g., with immortal GT1-7 cells in a dose- and time-dependent manner [64,65,66,67]. Additional mechanistic studies are now needed to better clarify the possible detrimental effects of Zn at the injury site of TSCI.

Furthermore, Zn plays a crucial role in the regulation of Brain-Derived Neurotrophic Factor (BDNF), which is considered to be one of the most important factors after TSCI concerning neuroregeneration [57,58,68]. In 2011, Wang et al. investigated the expression of Zn transporter 1 (ZnT-1) and BDNF along with Zn concentrations in spinal cord-injured rats. They found significantly decreased serum Zn levels in the SCI model but slightly increased Zn concentrations within the spinal cord. Further, the mRNA levels of ZnT-1 and BDNF were significantly increased in the SCI model group, indicating a physiological co-regulation [57].

In line with our results, McLain et al. found decreased serum Zn concentrations at admission in head trauma patients, which was combined with elevated urinary Zn losses [21]. In the current study, the Zn serum levels in group S dropped initially and reached their minimum beneath the reference level 9 h after trauma. Decreasing Zn levels after TSCI might be merely explained by Zn relocation from peripheral blood to the central nervous system to provide sufficient levels directly at the lesion site. Urinary Zn losses and consuming inflammatory processes might contribute further to decreasing Zn levels after trauma. Furthermore, the proliferation of many pathogens is Zn-dependent, likely constituting the underlying driving force for the negative acute phase regulation of plasma Zn concentration in infection [69].

In the current study, we demonstrate a particularly strong decrease in serum Zn concentration in the early phase after TSCI, which provides prognostic information on the probability of a neurological impairment via logistic regression modeling. Our data indicate that the changes are dynamic, and the timing of serum sampling and analysis is of key importance. The final model, including the Zn concentration difference from admission to 9 h after injury, yielded an AUC of 82.2% (CI: 64.0% to 100.0%) in relation to the occurrence of a neurological deficiency. Therefore, the probability of assigning a higher model-based score to patients with neurological impairment than to recovering patients is 82.2%, which may help in choosing the most promising therapeutic measures. Thus, we can assess a reasonable belief from the statistical perspective that these findings potentially better fit the criteria for a biomarker in clinical practice compared to former approaches [70]. In addition, our data support the concept of early adjuvant nutritional support in order to avoid a strong Zn deficiency, which may constitute an easily avoidable factor impairing successful neuronal regeneration. The present findings contribute to ongoing progress in the field of neurotrauma through the identification of suitable biomarkers with a potential to impact clinical practice in the future via differential immune manipulation based on Zn metabolism in patients with TSCI. Additionally, these attempts might profit from recent developments in the field of biomedical engineering. Real-time diagnostics strategies, such as real-time optical imaging, will enhance the possibilities of the live monitoring of metabolic processes within the inflammatory response to the injury [71], while proteomic profiling reveals promising approaches regarding the identification of reliable biosignatures for TBI [72] that might be efficiently transferable to TSCI.

### 4.1. Limitations

Despite its relevant findings, the current study is not free from limitations. Firstly, the sizes of the entire biobank established over recent years and the resulting patient groups are relatively small, especially when compared to other types of human injuries. However, within the field of TSCI research, the infrequency of this debilitating injury and the acute emergency and immediate intensive medical care are a key challenge to any scientific approach of collecting samples and enrolling patients into an analytical study. Besides a thorough collection and meticulous establishment of a biobank, the only other way to obtain a sufficiently high number of samples would be by international collaborations to overcome this obstacle [73]. However, especially in the field of trace elements, different populations often differ grossly in their basal states, making cross-country comparisons and analyses particularly challenging. Nevertheless, our limited approach yielded a promising model for the early detection of patients with a high risk of a neurological impairment after TSCI, which still needs to be replicated independently. A second major limitation relates to the biomarker, i.e., the serum Zn concentrations, which may not necessarily reflect the intracellular and physiologically relevant Zn status but rather constitute a regulated surrogate marker [74]. Lastly, the data acquired are from an observational study only and are not suitable for deducing mechanistic insights. However, the data support current concepts aiming at actively correcting micronutrient deficiencies by several measures to support regeneration after traumatic injuries. Respective future studies are needed to investigate the effects and health potential of active Zn supplementation for neurological health and recovery from severe disease. Considering the possible synergistic benefit from more than one biomarker in the final prognostic model, a systematic review and network metanalysis bear the potential to create a prognostic model with a chance of future clinical application. The interval from injury to the final neurological assessment after three months was chosen in the present study in view of the vast majority of patients recovering within the first three months following tetraplegia [75]. However, as there is level 2 evidence reporting that stability of neurological outcome is reached within a year from the time of injury [76,77], future studies evaluating the predictors of functional outcome 12 months post injury are required to assess the characteristics of recovery after 3 months.

### 4.2. Strengths

This study provides a detailed insight into the Zn concentration dynamics in the acute phase of patients with TSCI. It highlights the most informative time point and strategy for assessing the risk of neurological impairment. Importantly, the analysis of Zn concentrations was performed in a laboratory remote from the clinics, and the staff were blinded to the patients’ characteristics to reduce the potential for experimental bias. Given the rarity of this disease, it is a particular strength of the current study that blood was sampled frequently in a standardized manner, and that the samples were collected and stored safely to cover an exhaustive time span after the time of injury and to monitor the relevant kinetics.

## 5. Conclusions

Our results indicate that the analysis of the peripheral trace element Zn is an appropriate concept for assessing the risk of neurological impairment in unresponsive patients suspected of having a TSCI. Based on the regression model, including the difference in Zn concentration from admission to 9 h after injury, the overall chance of assigning a higher model-based score to patients with a neurological impairment is 82.2%. Monitoring trace element changes may contribute to a better understanding of the pathophysiology of TSCI and may strengthen the concept of adjuvant treatment options during the care of patients in the ICU.

## Figures and Tables

**Table 1:** Demographic and clinical characteristics of subjects. Abbreviations: NLI = Neurological Level of Injury; AO = AO-Classification; AIS = ASIA (American Spinal Injury Association) Impairment Scale. Age is expressed as median/mean years with their corresponding IQR (Interquartile Range) and 95% CI (Confidence Interval). Neurological remission was defined as an improvement in AIS within 3 months after the trauma.

Variable	All (*N* = 42)	G0 (*N* = 15)	G1 (*N* = 18)	C (*N* = 9)	*p*-Value
**Sex**					0.56
female	12 (29)	2 (13)	5 (28)	5 (56)	
male	30 (71)	13 (87)	13 (72)	4 (44)	
**Age**					0.17
min	15	20	15	27	
max	78	78	75	71	
median (IQR)	42.00 (27.75, 59.00)	49 (35.50, 62.00)	37.50 (21.25, 57.00)	40 (32.00, 59.00)	
mean (95% CI)	44.07 (38.35, 49.80)	48.60 (38.77, 58.43)	39.33 (30.18, 48.48)	46.00 (35.66, 56.34)	
**Etiology**					0.74
Fall from height	21 (50)	6 (40)	9 (50)	6 (67)	
Motor vehicle collision	16 (38)	6 (40)	7 (39)	3 (33)	
Other accident	5 (12)	3 (20)	2 (11)	0 (0)	
**AO**					0.17
A	29 (69)	8 (53)	15 (83)	6 (67)	
B	6 (14)	2 (13)	1 (6)	3 (33)	
C	7 (17)	5 (33)	2 (11)	0 (0)	
**NLI**					0.04
none	9 (21)	0 (0)	0 (0)	9 (100)	
cervical	14 (33)	4 (27)	10 (56)	0 (0)	
thoracical	14 (33)	10 (67)	4 (22)	0 (0)	
lumbar	5 (12)	1 (7)	4 (22)	0 (0)	
**AIS initial**					< 0.01
A	15 (36)	12 (80)	3 (17)	0 (0)	
B	7 (17)	1 (7)	6 (33)	0 (0)	
C	10 (24)	1 (7)	9 (50)	0 (0)	
D	1 (2)	1 (7)	0 (0)	0 (0)	
E	9 (21)	0 (0)	0 (0)	9 (100)	
**AIS final**					< 0.01
A	12 (29)	12 (80)	0 (0)	0 (0)	
B	3 (7)	1 (7)	2 (11)	0 (0)	
C	6 (14)	1 (7)	5 (28)	0 (0)	
D	12 (29)	1 (7)	11 (61)	0 (0)	
E	9 (21)	0 (0)	0 (0)	9 (100)	

**Table 2:** The American Spinal Injury Association Impairment Scale (AIS). AIS grades from A to E considering the completeness of paralysis and the motor and sensory function test [36].

AIS Grade	A	B	C	D	E
**Clinical State**	*Complete* no motor or sensory function is preserved in the sacral segments S4–S5	*Incomplete* sensory but not motor function is preserved below the NLI and includes the sacral segments S4–S5	*Incomplete* motor function is preserved below the NLI, and more than half of key muscles below the NLI have a muscle grade less than 3	*Incomplete* motor function is preserved below the NLI, and at least half of key muscles below the NLI have a muscle grade of 3 or more	*Normal* motor and sensory function is normal

**Table 3:** Specifics of the predictive models. For each model, included variables estimates are provided with their corresponding CI in brackets and the calculated Cohen’s d, while the interpretation is indicated below [46].

Variable	Model 1	Model 2	Model 3	Cohen’s d
(Intercept)	1.37 **	1.15 *	1.20 *	
	[0.50, 2.24]	[0.22, 2.09]	[0.02, 2.38]	
Zn 0 h (µg/L)	0.68			−0.632
	[−0.27, 1.63]			moderate
Zn 9 h (µg/L)		−0.75		0.636
		[−1.69, 0.20]		moderate
Zn 0 h–9 h (µg/L)			1.28	−1.12
			[−0.07, 2.63]	large
n	36	27	21	
AIC	39.88	32.16	24.45	
Pseudo-R² (Cragg-Uhler)	0.09	0.14	0.29	

** *p* < 0.01; * *p* < 0.05.

**Table 4:** Comparison of the corresponding demographics to the cervically injured patients reported by Kijima et al., 2019 [23]. The Kruskal-Wallis test for comparison of two independent samples and the Fisher’s exact test were used to assess the differences in numeric and categorical variables.

Variable	Data of the Current Study (*N* = 14)	Kijima et al., 2019 (*N* = 38) [23]	*p*-Value
**Sex**			> 0.99
female	3 (21.4%)	10 (26.3%)	
male	11 (78.6%)	28 (73.7%)	
**Age**			< 0.01
min	15.00	18.00	
max	77.00	85.00	
median (IQR)	47.50 (15.00, 77.00)	67.50 (18.00, 85.00)	
mean (95% CI)	45.43(33.98, 56.88)	63.579 (57.61, 69.55)	
**Etiology**			0.23
Fall from height	4 (28.6%)	15 (39.5%)	
Motor vehicle collision	8 (57.1%)	12 (31.6%)	
Other accident	2 (14.3%)	11 (28.9%)	
**AIS initial**			0.18
A	4 (28.6%)	13 (34.2%)	
B	4 (28.6%)	3 (7.9%)	
C	5 (35.7%)	10 (26.3%)	
D	1 (7.1%)	8 (21.1%)	
E	0 (0.0%)	4 (10.5%)	
**AIS final**			0.79
A	3 (21.4%)	6 (15.8%)	
B	1 (7.1%)	3 (7.9%)	
C	3 (21.4%)	8 (21.1%)	
D	7 (50.0%)	17 (44.7%)	
E	0 (0.0%)	4 (10.5%)	
